# Jovem de 17 Anos com Atresia Pulmonar e Septo Ventricular Íntegro, Submetido à Operação de Fontan e com Persistência da Fístula Coronário-Cavitária

**DOI:** 10.36660/abc.20201011

**Published:** 2021-06-08

**Authors:** Edmar Atik

**Affiliations:** 1 Universidade de São Paulo Hospital das Clínicas Faculdade de Medicina Universidade de São Paulo São PauloSP Brasil Instituto do Coração do Hospital das Clínicas da Faculdade de Medicina da Universidade de São Paulo , São Paulo , SP - Brasil

**Keywords:** Cardiopatias Congênitas, Cirurgia de Fontan, Atresia Pulmonar, Cardiomegalia, Ventrículo Direito/anormalidades, Arritmias Cardíacas, Fístula coronário-cavitária

## Introdução

Atresia pulmonar com septo ventricular íntegro constitui-se em anomalia congênita com interrupção total do fluxo de sangue entre o ventrículo direito e o tronco pulmonar, e em geral não é acompanhada de defeitos associados, exceto pela comunicação interatrial, presente em 20%, com predomínio do forame oval patente. ^1^ Decorre a atresia valvar da falta de desenvolvimento embriológico dessa estrutura fibrosa, podendo se localizar a nível valvar (membrana fibrosa), mas também a nível infundibular (fundo cego). Como consequências, surgem hipertrofia miocárdica e hipoplasia de ventrículo direito, hipoplasia do anel e da valva tricúspide, insuficiência tricúspide discreta e fluxo pulmonar dependente do canal arterial. Em vista da maior hipertensão em ventrículo direito, formam-se através de sinusóides conexões diretas com a circulação coronária, com fluxo em direção à aorta. Diz-se nessa situação que a circulação coronária é dependente do ventrículo direito e, quando se apresenta em grande magnitude, predispõe ao infarto do miocárdio, a arritmias, e também a sobrecarga volumétrica ventricular direita, por fluxo retrógrado da aorta. ^2^

Em casos nos quais a atresia valvar se desenvolve mais tardiamente no feto, a cavidade de ventrículo direito pode estar bem formada com suas três porções, de via de entrada, a trabecular e a da via de saída e, como consequência, ocorre insuficiência tricúspide acentuada, até com alteração tipo Ebstein da valva redundante e mixomatosa, adelgaçamento da parede ventricular, disfunção ventricular e insuficiência cardíaca direita, esta sobreposta à hipóxia. Em geral, não acompanha outros defeitos associados, e as artérias pulmonares se mostram de tamanho adequado. Nesses casos, não há a formação de conexão do ventrículo direito com as artérias coronárias por sinusóides.

### Como se exterioriza e evolui

No primeiro tipo, com hipoplasia de ventrículo direito, o quadro clínico se expressa precocemente na vida com hipóxia variável e de intensidade dependente da funcionalidade do canal arterial. A semiologia clínica se mostra com sopro contínuo suave na área pulmonar, segunda bulha hipofonética, sobrecarga de ventrículo esquerdo no eletrocardiograma, mas sem bloqueio divisional anterossuperior esquerdo, além de coração com dimensões próximas do normal. No tipo II, com maior insuficiência tricúspide e cavidade ventricular dilatada, sobrepõe-se à hipóxia quadro de insuficiência cardíaca direita com hepatomegalia. Há a demonstração clínica da cardiomegalia por impulsões sistólicas nítidas no precórdio, sopro intenso sistólico da insuficiência tricúspide, sobrecarga biventricular no eletrocardiograma, do tipo diastólica, e cardiomegalia à custa das cavidades direitas.

A evolução se mostra sempre desfavorável e em poucos dias, em ambos os tipos, na dependência da diminuição progressiva ou mesmo súbita do canal arterial, assim como do grau da insuficiência tricúspide e da disfunção do coração direito.

### Como se trata

**Clínica:** Como em ambos os tipos de atresia pulmonar — aquele com ventrículo direito hipoplásico e o com ventrículo direito dilatado — há a dependência do canal arterial para a funcionalidade da circulação pulmonar. Portanto, o uso de prostaglandina E1 torna-se imprescindível. Na insuficiência cardíaca direita associada, medidas restritivas de volume são também empregadas, mesmo com o uso de diuréticos, mas sempre com a devida cautela, em face da hipoxemia.

**Cirúrgica:** Anastomose sistêmico pulmonar tipo *Blalock-Taussig* se impõe no primeiro tipo, no qual a hipóxia precisa ser minimizada prontamente. Em casos nos quais o ventrículo direito se mostra bem formado, especialmente com maior dilatação e além da continuidade das estruturas da via de saída do ventrículo direito e do tronco pulmonar, a restituição do fluxo entre essas estruturas se torna possível por atuação por cateteres, que perfuram a valva com atresia por radiofrequência. A circulação coronária dependente do ventrículo direito é em geral preservada, exceto quando há fluxo da esquerda para a direita, funcionando como fístulas arteriovenosas, mas de grande monta.

### Como evolui após a operação

O controle da hipóxia é melhor obtido que o da insuficiência tricúspide, especialmente quando a mesma se mostra acentuada. Em evolução posterior, operação tipo Fontan se realiza em época oportuna, precedida inicialmente pela técnica de Glenn. Na possibilidade de restituição do fluxo pulmonar, após conexão direta das estruturas direitas, observa-se evolução mais favorável, exceto pelo aparecimento da insuficiência valvar pulmonar, que pode necessitar de reparo evolutivo.

O propósito dessa avaliação é a demonstração da evolução favorável após a operação de Fontan em pacientes nos quais permanece a fístula coronário-cavitária entre o ventrículo direito e a artéria coronária esquerda, desde que seja de discreta repercussão.

### Descrição do caso clínico

**Dados clínicos** : Logo ao nascer, desenvolveu quadro hipóxico acentuado que requereu a feitura de anastomose de Blalock-Taussig com 2 dias de vida. Com 12 meses, realizada operação de *Glenn* bidirecional, e com 5 anos, completou o princípio Fontan, com tubo externo fenestrado. Desde então, se mantém sem sintomas, em uso de warfarina, com saturação de oxigênio de 88%. Sopro sistólico e diastólico o acompanha desde o início, decorrente de fístula coronário-cavitária persistente entre o ventrículo direito e a artéria descendente anterior, com fluxo bidirecional.

Exame físico: Eupneica, acianótica, pulsos normais, sem turgência jugular. Peso: 58 kg; altura: 163 cm; pressão arterial (PA): 90/60 mm Hg; frequência cardíaca (FC): 74 bpm; saturação de oxigênio: 88%. Aorta não palpada na fúrcula.

No precórdio, *ictus cordis* no 4 ^o^ espaço intercostal esquerdo e impulsões sistólicas discretas na borda external esquerda (BEE). Bulhas cardíacas hiperfonéticas; sopro sistólico, ++/4, rude, e sopro diastólico suave ++/4 na BEE baixa e pouco irradiado. O fígado não era palpado e pulmões limpos.

### Exames Complementares

**Figura 1:**
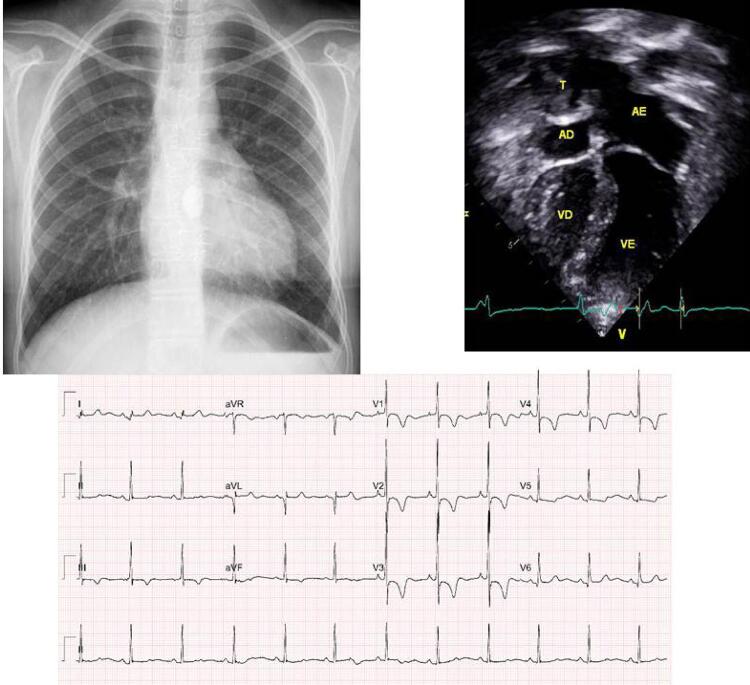
– Radiografia de tórax mostra área cardíaca normal, arco médio retificado e trama vascular pulmonar normal. Eletrocardiograma salienta sobrecarga ventricular direita com complexo Rs em V1 e ondas T negativas de V1 a V5. Ecocardiograma apical de 4 câmaras salienta a hipoplasia de ventrículo direito com septo ventricular abaulado para a esquerda, com cavidades cardíacas normais além do tubo (t) de fenestração intraatrial direito. AD: átrio direito; AE: átrio esquerdo; VD: ventrículo direito; VE: ventrículo esquerdo.

**Eletrocardiograma** mostrava ritmo sinusal e sinais de sobrecarga de ventrículo direito com complexo Rs em V1 e onda T negativa de V1 a V5. Os potenciais de ventrículo esquerdo eram salientes com complexos qRs em precordiais esquerdas. Sem sinais de sobrecargas atriais. AQRS: +80 ^o^ , AT: -30 ^o^ , AP: +30 ^o^ ( Figura 1 ).

**Radiografia de tórax** mostra área cardíaca normal (índice cardiotorácico: 0,46) com arco ventricular saliente, arco médio retificado e trama vascular pulmonar normal ( Figura 1 ).

**Ecocardiograma** mostrou bom funcionamento da operação cavopulmonar. As veias cavas, inferior e superior, com fluxos laminares em velocidade de 0,38 m/s; tubo externo para artéria pulmonar direita com velocidade de 0,46 m/s. O fluxo da fenestração era dirigido para o átrio direito com velocidade de 1,04 m/s. O ventrículo direito era hipoplásico com septo ventricular desviado para a direita com ventrículo esquerdo levemente hipertrófico e dilatado, com função normal de 60% pelo método de Simpson. Havia uma fístula entre o ventrículo direito e a artéria descendente anterior, de pequena dimensão, com fluxo bidirecional ( Figura 1 ).

**Figura 2:**
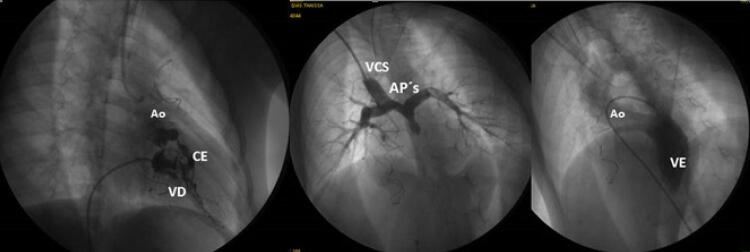
– Angiocardiografia cardíaca antes da operação de Fontan mostra a boa funcionalidade do Glenn bidirecional em B, com ventrículo esquerdo rechaçado pela maior pressão de ventrículo direito em C, e a fístula coronário-cavitária do ventrículo direito hipertrófico e hipoplásico para a artéria coronária esquerda e aorta, em A. AO: aorta; VD: ventrículo direito; VE: ventrículo esquerdo; CE: artéria coronária esquerda; AP´s: artérias pulmonares; VCS: veia cava superior.

**Cateterismo cardíaco** realizado antes da operação de Fontan salientava a boa funcionalidade do Glenn bidirecional com ventrículo esquerdo rechaçado pela maior pressão de ventrículo direito e a fístula coronário-cavitária do ventrículo direito hipertrófico e hipoplásico para a artéria coronária esquerda e aorta ( Figura 2 ).

### Diagnóstico Clínico

Atresia pulmonar com septo ventricular íntegro com ventrículo direito hipoplásico e fístula persistente coronário-cavitária entre o ventrículo direito e a artéria coronária esquerda em jovem de 17 anos, em evolução após 11 anos da operação de Fontan.

### Características Clínicas

- **Raciocínio Clínico:** a evolução da operação de Fontan cursa em geral sem sopros cardíacos e com alguma limitação física, em face da diminuição do débito cardíaco. Na ausculta cardíaca desse paciente, o sopro sistólico e diastólico chamou muito a atenção clínica e a primeira suposição com diagnóstico prévio da atresia pulmonar com septo ventricular íntegro foi de fístula coronário-cavitária em ventrículo direito, que persistia desde o nascimento. A maior pressão do ventrículo direito orienta a passagem do sangue em direção à artéria coronária durante a sístole ventricular (sopro sistólico) e, ao contrário, na diástole, quando o sangue da aorta se dirige ao próprio ventrículo direito (sopro diastólico). Os exames complementares salientaram a presença de sobrecarga do ventrículo direito no eletrocardiograma, decorrente da sobrecarga diastólica imposta pela fístula coronário-cavitária, mas não o suficiente para causar dilatação do ventrículo direito. Daí, pode-se concluir que essa fístula não causou sobrecarga anatomofuncional que influísse na dinâmica circulatória.
- **Diagnóstico Diferencial:** raramente após a operação de Fontan se ausculta sopro sistólico e diastólico, a não ser em situações inusitadas como em dupla lesão valvar aórtica associada, por exemplo. Mas, nessa situação, a repercussão clínica passa a ser desfavorável em face do aumento retrógrado da pressão arterial pulmonar. Essa mesma ausculta pode ainda ocorrer em presença de lesão de uma das valvas atrioventriculares, com predomínio da estenose, que por sua vez também causa problemas evolutivos, da mesma maneira. Assim, considerando o achado de sopro sistólico e diastólico neste paciente após a operação de Fontan, fístula coronário-cavitária seria a única causa compatível com a boa evolução.

**Conduta:** Em face da evolução favorável do paciente dada a pequena repercussão clínica da fístula coronário-cavitária, a conduta expectante fora tranquilamente continuada, ao lado da medicação anticoagulante preconizada.

## Discussão

Embora seja paliativa a operação de Fontan, com complicadores evolutivos, continua a oferecer boas perspectivas desde que obedeça rigorosamente aos critérios de indicação. Na presença conhecida da fístula coronário-cavitária e em associação da atresia pulmonar com septo ventricular íntegro, não se cogitou daí o seu fechamento em face da discreta repercussão da mesma, não salientando assim consequências desfavoráveis, e ainda por se situar no mesmo sistema sanguíneo arterial, sem interferência no sistema venoso. Na hipótese de a fístula mostrar maior repercussão, a conduta de fechamento da mesma obrigatoriamente deve ser indicada, por ocasião da operação de Fontan. Nessa ocasião, *a* lternativa de fechamento da valva tricúspide também resulta adequada no sentido de tornar a fístula com menor repercussão dinâmica. Tal conduta é adotada por ocasião da feitura da operação de Fontan ou mesmo em período prévio. ^3^ Tal procedimento se torna necessário antes da operação de Fontan, em vista da conhecida mortalidade de pacientes com circulação arterial das artérias coronárias dependentes do ventrículo direito. ^4 , 5^ Ela alcançou, segundo Calder, ^5^ cifra de 40% (47 de 116 pacientes) principalmente relacionados a interrupções e estenoses coronárias. Salienta-se que a presença de fístulas coronário-cavitárias, por si só, não é responsável pela mortalidade, exceto com lesões obstrutivas arteriais associadas e com fístulas de grande tamanho.

Na literatura, há poucos artigos correlacionando a operação de Fontan com fístulas coronário-cavitárias persistentes. Cheung ^6^ encontrou isquemia miocárdica em 2 dos 4 casos com persistência de fístulas coronário-cavitárias após Fontan. Por outro lado, Guleserian ^7^ verificou boa evolução dos 19 pacientes com fístulas coronário-cavitárias submetidos à operação de Fontan e em 7 casos, após Glenn. A sobrevida desses pacientes correspondeu a 81,3% em 5, 10 e 15 anos após Fontan, com sobrevida média de 12,1 anos. Salienta ainda esse autor que a mortalidade se restringiu aos pacientes com quadro isquêmico mais exuberante (6 dos 32–18,8%), mas em período precoce, de apenas 3 meses após o Blalock-Taussig. Nesse grupo, havia atresia aortocoronária em 3 desses pacientes.

No entanto, evolução mais desfavorável foi relatada por Elias, ^8^ em vista da mortalidade de 9% (11/120 pacientes) em período evolutivo de 9,1 anos após Fontan; morte súbita ocorreu em 6 dos 11 pacientes, e destes, 4 tinham circulação coronária dependente do ventrículo direito. A causa da morte desses pacientes se relacionou com isquemia miocárdica.

Em suma, pode-se concluir que pacientes com fístulas coronário-cavitárias de repercussão devam ser reparados precocemente e os submetidos ao princípio Fontan, mesmo de menor repercussão, devem ser monitorados e investigados por testes de esforço em acompanhamento rigoroso. ^9^
