# Supplementary material for: Jovem de 17 Anos com Atresia Pulmonar e Septo Ventricular Íntegro, Submetido à Operação de Fontan e com Persistência da Fístula Coronário-Cavitária
Source: Arq Bras Cardiol. 2021 Jun 8;116(6):1161–4. [Article in Portuguese] doi: 10.36660/abc.20201011 (PMC8288529; doi:10.36660/abc.20201011)
Supplement: Translation

# Translation: 17-Year-Old Man with Pulmonary Atresia and Intact Ventricular Septum Submitted to Fontan Operation, and with Persistent Coronary-Cavitary Fistula

**Authors:** Edmar Atik

**Affiliations:** 1 Brasil Instituto do Coração do Hospital das Clínicas da Faculdade de Medicina da Universidade de São Paulo, São Paulo, SP - Brasil

**Keywords:** Heart Defects Congenital, Fontan Surgery, Pulmonary Atresia/surgery, Cardiomegaly, Right Ventricle/Abnormalities, Arrhythmias, Cardiacs, Coronary-cavitary fistula

## Introduction

Pulmonary atresia with intact ventricular septum is a congenital anomaly with total interruption of blood flow between the right ventricle and the pulmonary trunk, and in general it is not accompanied by associated defects, except for interatrial septal defect, present in 20%, with predominance of patent foramen ovale.^1^ Valve atresia results from the lack of embryological development of this fibrous structure, which can be located at the valve level (fibrous membrane) but also at the infundibular level (blind fundus). As a consequence, myocardial hypertrophy and hypoplasia of the right ventricle, hypoplasia of the ring and tricuspid valve, mild tricuspid insufficiency and pulmonary flow dependent on the ductus arteriosus appear. In view of greater hypertension in right ventricle, direct connections with the coronary circulation are formed through sinusoids, with flow towards the aorta. In this situation, it is said that the coronary circulation is dependent on the right ventricle, and when these connections are present in great magnitude, they predispose to myocardial infarction, arrhythmias and right ventricular volume overload, due to retrograde flow from the aorta.^2^

In cases where valve atresia develops later in the fetus, the right ventricular cavity may be well formed with its three portions, the entrance route, the trabecular and the exit route and, as a consequence, there is marked tricuspid insufficiency, even with Ebstein-type alteration of the redundant and myxomatous valve, ventricular wall thinning, ventricular dysfunction and right heart failure, the latter superimposed on hypoxia. In general, it is not accompanied by other associated defects and the pulmonary arteries are of adequate size. In these cases, there is no connection between the right ventricle and the coronary arteries by sinusoids.

### How it exteriorizes and evolves

In the first type, with hypoplasia of the right ventricle, the clinical condition is expressed early in life with variable hypoxia and intensity dependent on the functionality of the ductus arteriosus. Clinical semiology is shown with a smooth continuous murmur in the pulmonary area, second heart sound of diminished intensity, left ventricular overload on the electrocardiogram, but without left anterior and superior divisional block, in addition to a heart with dimensions close to normal. In type II, with greater tricuspid regurgitation and dilated ventricular cavity, hypoxia is associated with right heart failure with hepatomegaly. There is a clinical demonstration of cardiomegaly by clear systolic impulses in the precordium, intense systolic murmur of tricuspid regurgitation, diastolic biventricular overload on the electrocardiogram, and cardiomegaly at the expense of the right cavities.

Evolution is always unfavorable and takes a few days, in both types, depending on progressive or even sudden ductus arteriosus decrease, degree of tricuspid insufficiency and right heart failure.

### How it is treated

**Clinical treatment:** As in both types of pulmonary atresia, the one with a hypoplastic right ventricle and the one with an enlarged right ventricle, there is dependence on the arterial ductus for pulmonary circulation functionality, and the use of prostaglandin E1 becomes essential. In overlying right heart failure, restrictive volume measures are also used, even with the use of diuretics, but always with caution, due to hypoxemia.

**Surgical treatment:** Blalock-Taussig pulmonary systemic anastomosis in the first type in which hypoxia needs to be minimized promptly. In cases in which the right ventricle is well formed, especially with greater dilation and in addition to the continuity of the structures of the right ventricular outflow tract and the pulmonary trunk, the flow between these structures is made possible by actuation through catheters piercing the atretic valve with radio frequency. Coronary circulation, dependent on the right ventricle, is generally preserved, except when there is greater flow from left to right, functioning as arteriovenous fistulas, of great magnitude.

### How it evolves after the operation

Control of hypoxia is better achieved than that of tricuspid regurgitation, especially when it is marked. In a later evolution, Fontan operation is performed on a timely manner, initially preceded by the Glenn technique. In the possibility of restitution of the pulmonary flow, after direct connection of the right structures, a more favorable evolution is observed, except for the appearance of pulmonary valve insufficiency, which may require evolutionary repair.

The purpose of this evaluation is to demonstrate the favorable evolution after Fontan operation in patients in whom the coronary-cavitary fistula remains between the right ventricle and the left coronary artery, as long as it has a slight repercussion.

### Description of the Clinical Case

**Clinical data:** Right after birth, the patient developed a severe hypoxic condition that required Blalock-Taussig anastomosis to be performed at 2 days of age. After 12 months, bidirectional Glenn operation was performed and, at the age of 5, the patient completed the Fontan principle, with a fenestrated external tube. Since then, patient has remained symptom-free, using warfarin, with oxygen saturation of 88%. Systolic and diastolic murmur accompanies it from the beginning, due to persistent coronary-cavitary fistula between the right ventricle and the anterior descending artery, with bidirectional flow.

**Physical examination:** Eupneic, acyanotic, normal pulses, no jugular turgency. Weight: 58 kg; height: 163 cm; BP: 90/60 mm Hg; HR: 74 bpm, oxygen saturation = 88%. Aorta not palpated in the suprasternal notch.

In the precordium, apical impulse in the 4^th^left intercostal space and discrete systolic impulses at the left external border. Accentuated heart sounds; systolic murmur, ++/4 intensity, rough, and mild diastolic murmur ++/4, along the left sternal border. The liver was not palpable and the lungs were clean.

### Complementary Exams

**Electrocardiogram** showed sinus rhythm and signs of right ventricular overload with Rs complex in V1 and negative T wave from V1 to V5. Left ventricular potentials were prominent with qRs complex in left precordial leads. No signs of atrial overload. AQRS: + 80^o^, AT: -30^o^, AP: + 30^o^( Figure 1 ).

**Figure 1:** – Chest X-ray shows normal cardiac area, rectified medium arch and normal pulmonary vascular network. Electrocardiogram highlights right ventricular overload with Rs complex in V1 and negative T waves from V1 to V5. Apical 4-chamber echocardiogram highlights right ventricular hypoplasia with ventricular septum deviated to the left side, with normal cardiac cavities in addition to the right intraatrial fenestration tube (t). RA: right atrium; LA: left atrium; RV: right ventricle; LV: left ventricle.

**Chest radiography** shows normal cardiac area (cardiothoracic index: 0.46) with protruding ventricular arch, rectified medium arch and normal pulmonary vascular network ( Figure 1 ).

**Echocardiogram** showed good functioning of the cavopulmonary operation. Inferior and superior vena cava, with laminar flows at a speed of 0.38 m/s; external tube for right pulmonary artery with a speed of 0.46 m/s. The fenestration flow was directed to the right atrium at a speed of 1.04 m/s. The right ventricle was hypoplastic with ventricular septum deviated to the right with a slightly hypertrophic and dilated left ventricle, with normal function of 60% using the Simpson method. Fistula between the right ventricle and the small anterior descending artery, with bidirectional flow ( Figure 1 ).

**Cardiac catheterization** performed before the Fontan operation highlighted the good functionality of the bidirectional Glenn, with left ventricle compressed by the higher pressure of the right ventricle, and coronary-cavitary fistula of the hypertrophic and hypoplastic right ventricle to the left coronary artery and aorta ( Figure 2 ).

**Figure 2:** – Cardiac angiocardiography before the Fontan operation shows the good functionality of the bidirectional Glenn in B, with the left ventricle rejected by the higher pressure of the right ventricle in C, and the coronary-cavitary fistula of the hypertrophic and hypoplastic right ventricle to the left coronary artery and aorta, in A. AO: aorta; RV: right ventricle; LV: left ventricle; LCA: left coronary artery; PA´s: pulmonary arteries; SVC: superior vena cava.

**Clinical Diagnosis:** Pulmonary atresia with intact ventricular septum with hypoplastic right ventricle and persistent coronary-cavitary fistula between the right ventricle and the left coronary artery in a 17-year-old man, evolving 11 years after the Fontan operation.

### Clinical Characteristics

- **Clinical Reasoning:** Evolution of Fontan operation is generally without heart murmurs and with some physical limitation due to decreased cardiac output. In the cardiac auscultation of this patient, systolic and diastolic murmur drew a lot of clinical attention and the first assumption with previous diagnosis of pulmonary atresia with intact ventricular septum was coronary-cavitary fistula in the right ventricle, which persisted since birth. Higher pressure in the right ventricle directs the passage of blood towards the coronary artery during ventricular systole (systolic murmur) and, in contrast, in diastole, when blood from the aorta goes to the right ventricle itself (diastolic murmur). Complementary exams highlighted the presence of right ventricular overload on the electrocardiogram, resulting from the diastolic overload imposed by the coronary-cavitary fistula, but not enough to cause right ventricular dilation. It can be concluded from this that this fistula did not cause anatomo-functional overload that would influence the circulatory dynamics.
- **Differential Diagnosis:** Rarely after the Fontan operation systolic and diastolic murmur is heard, except in unusual situations such as a double associated aortic valve injury, for example. But, in this situation, the clinical repercussion becomes unfavorable in view of the retrograde increase in pulmonary arterial pressure. The same auscultation can also occur in the presence of an injury to one of the atrioventricular valves, with predominance of stenosis, which in turn also causes evolutionary problems, in the same way. Thus, considering the finding of a systolic and diastolic murmur in this patient after the Fontan operation, coronary-cavitary fistula would be the only cause compatible with the good evolution.

**Management:** In view of the favorable evolution of the patient due to the small clinical repercussion of the coronary-cavitary fistula, the expected management was smoothly continued along with the recommended anticoagulant medication.

## Discussion

Although Fontan operation is palliative, with evolutionary complications, it continues to offer good prospects as long as it strictly complies with the indication criteria. In the known presence of coronary-cavitary fistula and in association of pulmonary atresia with intact ventricular septum, its closure was not considered, due to its slight repercussion, thus not highlighting unfavorable consequences, and also because it is located in the same arterial blood system, without interference with the venous system. In the event that the fistula shows greater repercussions, fistula closure must be indicated upon the Fontan operation. On this occasion, choosing to close the tricuspid valve is also appropriate to make the fistula less dynamic. This management is adopted upon the Fontan procedure or even before.^3^ Such procedure becomes necessary before Fontan, in view of the well-known mortality of patients with arterial circulation of right ventricle-dependent coronary arteries.^4 , 5^ According to Calder,^5^ mortality reached 40% (47 out of 116 patients), mainly related to interruptions and stenosis of the coronary arteries. It should be noted that the presence of coronary-cavitary fistulas, per se, is not responsible for mortality, except with associated arterial obstructive lesions and large fistulas.

There are few articles in the literature correlating the Fontan operation with persistent coronary-cavitary fistulas. Cheung^6^ found myocardial ischemia in 2 of the 4 cases with persistent coronary-cavitary fistulas after Fontan. On the other hand, Guleserian^7^ found a good evolution of the 19 patients with cavitary-coronary fistulas submitted to Fontan and in 7 cases after Glenn. The survival of these patients was 81.3% at 5, 10 and 15 years after Fontan, with an average survival of 12.1 years. This author also points out that mortality was restricted to patients with a more exuberant ischemic condition (6 out of 32–18.8%), but also in an early period, just 3 months after Blalock-Taussig. In this group, aortocoronary atresia was present in 3 of these patients.

However, more unfavorable evolution was reported by Elias,^8^ in view of the 9% mortality (11/120 patients) in an evolutionary period of 9.1 years after Fontan. In these patients, sudden death occurred in 6 of the 11 patients and, of these, 4 had coronary artery circulation dependent on the right ventricle. The cause of death of these patients was related to myocardial ischemia.

In summary, it can be concluded that patients with repercussion coronary-cavitary fistulas should be repaired early and those submitted to the Fontan principle, even with lesser repercussions, should be monitored and investigated by stress tests under strict evaluation.^9^
